# Motion artifact on computed tomography scan suggesting an unstable 3-column spine injury: case report of a "near miss" root cause of unneeded surgery

**DOI:** 10.1186/1754-9493-7-35

**Published:** 2013-11-25

**Authors:** Sunny H Patel, Timothy A Moore

**Affiliations:** 1 Department of Orthopaedic Surgery, University Hospitals Case Medical Center, 11100 Euclid Avenue, Cleveland, OH 44106, USA; 2 Departments of Orthopaedic Surgery and Neurosciences, Case Western Reserve University School of Medicine, MetroHealth Medical Center H910C, 2500 MetroHealth Drive, Cleveland, OH 44109, USA

**Keywords:** CT scan, Motion artifact, Lumbar spine, Three-column injury, Polytrauma, Physical examination

## Abstract

**Background:**

Polytrauma patients often present with altered mental status, thus making clinical examination challenging. Due to its reliability for detecting traumatic injuries to the spine, computed tomography (CT) is generally the imaging study of choice when the mechanism of injury and/or preliminary exam suggests spinal injury. However, motion artifact may lead to false diagnoses.

**Case report:**

A 19-year-old intoxicated female involved in a high-speed motor vehicle crash suffered multiple spine, head, chest, and abdominal injuries. CT scan also suggested an unstable three column ligamentous injury at L2-3. Preparations were made for surgery the following morning, by which time her mental status had improved. She was re-examined in the operating room prior to induction by anesthesia and no focal lumbar pain or tenderness was detected. Imaging was further reviewed and motion artifact at the L2-3 level was noted. The surgery was cancelled.

**Conclusion:**

Motion artifact mimicked an unstable three column ligamentous injury at the L2-3 level. Findings on CT scan should always be correlated to physical exam in order to avoid wrongful surgical intervention.

## Background

Helical computed tomography (CT) has rapidly replaced radiographs for evaluation of the thoracolumbar spine in blunt trauma patients. CT is especially useful for patients that cannot provide a reliable examination. Studies have shown CT to have sensitivity from 96-99% and specificity up to 99% in the detection of thoracolumbar injury [1-3].

However, the possibility of false positive results still exists. These errors are generally due to motion generated artifact. There have been multiple case reports of motion generated artifact in the cervical spine [4-6], but no such reports exist for the thoracolumbar spine. In this article, we present a case of motion artifact on a trauma series thoracolumbar CT that initially suggested an unstable three column ligamentous injury. The patient was scheduled to undergo operative stabilization until re-examination revealed no clinical signs of injury.

## Case report

**Figure 1:**
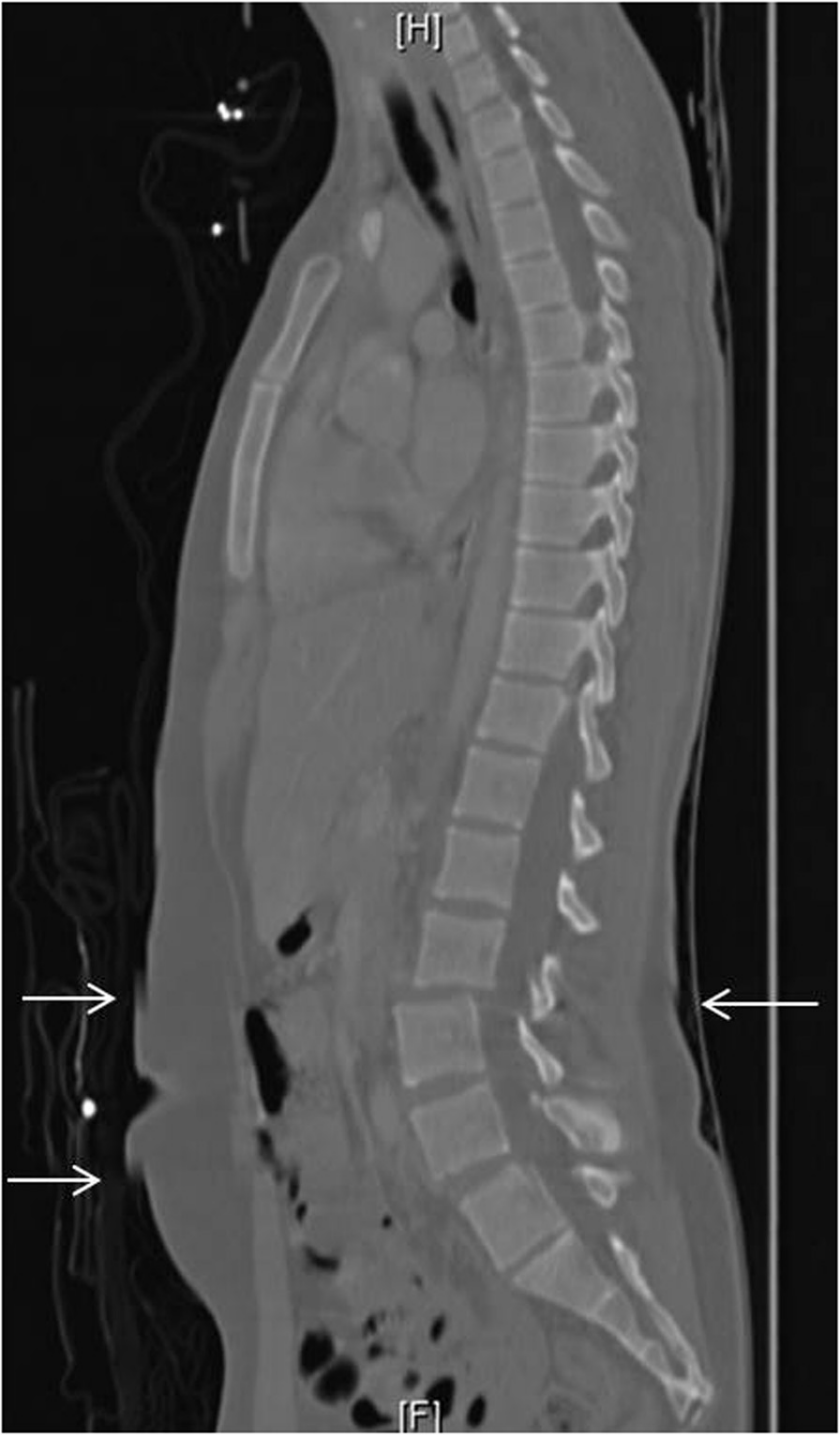
**Sagittal reconstruction of abdominal CT scan showing what appears to be a three-column ligamentous injury at L2-3.** However, motion artifact can be seen anteriorly and posteriorly (arrows).

A 19-year-old female was involved in a high-speed motor vehicle crash in which her car hit a tree. She was an unrestrained driver who was ejected from the vehicle. On arrival, the patient was amnestic to the event and was positive for alcohol use. Primary survey was significant only for agitation and confusion. Her Glasgow Coma Scale (GCS) score was 14 on arrival. Secondary survey did not reveal any focal tenderness or step-offs in her thoracolumbar spine. The patient was subsequently sedated. On further examination, she was moving all extremities spontaneously and had 4/5 strength in bilateral upper and lower extremities when challenged. The initial read on CT imaging showed what was thought to be a traumatic three column ligamentous injury resulting in a Grade 1–2 retrolisthesis of L2 on L3 (Figure 1). CT also demonstrated multiple other injuries including C6-T3 spinous process fractures, right lateral mass of C2 fracture, left temporal subarachnoid hemorrhage, grade 3 splenic laceration, grade 2 liver laceration, multiple bilateral rib fractures, small left pneumothorax, bilateral scapular fractures, right clavicle fracture, left mandibular fracture, and left sacral fracture. Given the unreliability of her exam and the presence of an injury mechanism that would allow for an unstable ligamentous injury at L2-3, preparations were made to perform a posterior spinal fusion the following morning.

At that time, the patient’s mental status had improved significantly. In the operating room just before induction by anesthesia,a repeat physical examination was performed. She was found to have no focal tenderness in her lumbar spine. The CT scan of her lumbar spine was further reviewed. Upon closer examination, there was a segment of motion artifact directly at the L2-L3 level evidenced by soft tissue abnormality located anteriorly and posteriorly. Imaging was reviewed with a radiologist, who agreed. The decision was made to cancel this surgery.

This case demonstrates the importance of clinical examination in evaluating acute spinal injury. The initial examination was unreliable, but not suggestive of a significant unstable injury. The patient should have been re-evaluated once the physical exam was reliable. This did not occur until the patient was in the operating room being prepared for surgery. Providers must rely on the physical examination with technology as an adjunct in evaluating and treating polytrauma patients with an altered mental status.

Holmes et al. suggest that pain and/or tenderness may be a sole predictor of thoracolumbar spinal injury. Additionally, while the incidence was low, the negative predictive values for pain and tenderness to palpation were 96% and 94%, respectively [7]. Studies have shown that 60-81% of patients with thoracolumbar spine injuries diagnosed by radiographs have thoracolumbar spine pain or tenderness [7-9].

Inaba et al. compared thoracolumbar clinical exam findings in blunt trauma patients with CT scan as the reference [10]. Their clinical examination consisted of assessment of skin and soft-tissue, presence of deformity, tenderness to palpation, and neurologic deficits. For assessment of fractures requiring surgical intervention, this clinical examination had a sensitivity and negative predictive value of 100%. However, a limitation of this study was a small number of cases requiring operative intervention. Although clinical examination alone is not sufficient in evaluating for thoracolumbar spinal injury, this data suggests that a lack of pain or tenderness to palpation in a reliable patient should warrant reassessment or further imagining prior to any surgical intervention.

This patient appeared to a have a three column ligamentous injury without any bony involvement. In a series of 24 patients with chance-type injuries, only 1 (4.2%) had a purely soft-tissue flexion distraction injury [11]. These results suggest that a flexion-distraction injury without fracture is a relatively rare occurrence. Given this case and these findings, a CT scan suggestive of a purely soft-tissue flexion-distraction injury should be scrutinized carefully.

Motion artifact generally occurs when the patient moves subtly during image acquisition, which would subsequently lead to an error during sagittal reconstruction. While the artifact is present at the affected spinal level, it is also present in the soft tissues anteriorly and posteriorly. Similar cases of motion artifact have been reported in the cervical spine. Sugimoto et al. described a case in which motion artifact appeared as a cervical dens fracture in a polytrauma patient with altered mental status who was involved in a motorcycle crash [4]. Sciubba et al. showed motion artifact related C5-6 subluxation in a patient with transient paresthesias and weakness of the upper extremities after a collision during a lacrosse game [5]. Daffner at al reported that motion artifact can mimic an avulsion fracture of the anterior aspect of the dens [6]. This appears to be the first reported case of motion artifact mimicking thoracolumbar spinal injury.

This case also emphasizes the importance of communication between services in the care of a polytrauma patient. The responsibility of this near “never event” lies with the consulting orthopaedic spine surgery team. However, a communication failure also occurred in the case example. The initial radiology read on the CT scan changed. At our institution, a radiology resident makes “preliminary” reads during the night that are occasionally amended by the attending radiologist the following day. The initial read was amended in this case, but there was no communication of the change made known to either the general surgery trauma team or the orthopaedic spine team.

## Conclusion

This is a case of motion artifact mimicking an L2-3 ligamentous injury after a motor vehicle crash. Initial clinical exam was unreliable due to patient intoxication and multiple other distracting injuries. Prior to induction by anesthesia, repeat clinical examination with normal patient mentation did not suggest thoracolumbar injury, and re-evaluation of the thoracolumbar CT scan showed motion artifact. This case highlights the importance of using imaging in conjunction with clinical examination to prevent unnecessary and/or harmful intervention. This case also demonstrates the role of communication in preventing “never events.” Additionally, all physicians involved in care of blunt trauma patients with suspected spinal injury should thoroughly review CT scans for soft tissue injury that may suggest motion artifact.

## Consent

Written informed consent was obtained from the patient for publication of this Case report and any accompanying images. No patient identifiers were used in this case report.

## Competing interests

The authors have no competing interests to declare in the preparation or finalization of this manuscript.

## Authors’ contributions

SHP was responsible for the initial draft, literature review, and revisions. TAM was responsible for concept/design, oversight, and revisions. All authors read and approved the final manuscript.
